# An Account of the Dandy, or Rheumatic Fever of the West Indies

**Published:** 1829-01-01

**Authors:** 


### DANDY, OR RHEUMATIC FEVER.

Several months ago we announced the visitation of this new disease—this NOVA PESTIS, which is ascribed, like the celebrated Bulam, to an African origin. To Dr. Stedman, of Santa Cruz, we are now indebted for a very detailed account of the disease, in the last Number of our

Northern contemporary. This curious disease first made its appearance in the Island and Town of St. Thomas, about the end of 1827, where it soon affected the whole of the inhabitants, 12,000 in number, not sparing even the medical practitioners of the place. It caused great alarm at first, and was considered as the plague; but it was soon discovered that the enemy was more painful than dangerous. The fever was readily divisible into three stages, each distinguished by very striking characters. The invasion of the symptoms was as sudden as their progress was rapid. A person walking in the street would be instantly seized with acute pain in one or both knees, ankles, wrists, or other, or all joints, though usually only in one, which would become suddenly so stiff, as to be incapable of motion without great pain. The most common mode of attack, however, was as follows:—a person in perfect health would suddenly feel a stiffness amounting almost to pain, in one of his fingers—frequently the little finger. The stiffness increasing, the pain would become intense, spreading rapidly over the hand, up the arm, and even to the shoulder. The fingers of both hands would, in a few hours, become swelled, stiff, and painful, preventing all motion. To these, in a short time, succeeded restlessness, depression of spirits, some nausea—and occasionally vomiting. Then came on shivering, fever, great heat of skin, intense head-ache, acute pain in the back, knees, ankles, and indeed all the joints. The most distressing and peculiar symptom was an intense pain in the eye-balls, which felt to the patient, as if too large for the orbit, and ready to burst from the head. As the fever or re-action became completely established, the whole body was racked with pain—the back and the eye-balls being most complained of, "in some, *the features were swollen and distorted, especially the eye-lids.*" Another peculiarity was, that during the existence of high fever, and excitement, the patients always felt intense coldness, and never could get enough of warm clothing. Those who had had other fevers of the country, declared that they never suffered any thing equal to this. These symptoms, if not relieved by art, continued from 24 to 36 hours, when the fever and pains abated, leaving the patient in a state of languor and irritability, for three days, during which, there was an interval of complete apyrexia, though the feelings were far from being in a state of comfort. Thus, the taste was lost, the appetite was deranged, &c. On the third or fourth day, the fever returned, in a greater or less degree; and, at this time, an efflorescence appeared on the palms of the hands, spreading gradually over the whole body, and bearing some resemblance to a mixture of measles and scarlet fever. This eruption was accompanied, in many cases, by swelling of the face, hands, and feet, and "distressing tingling,"* which changed into intolerable itching, that almost drove the sufferers mad. The efflorescence generally began to fade on the second day, and was gone by the third, followed by desquamation. This was the second, or eruptive stage—one which, in the author's opinion, stamped its character, and proved it to belong to the exanthemata. After this stage, the patient began to recover his spirits and strength—though the sense of taste, in many cases, continued absent. Many people did not get rid of the pains in their limbs and joints for several weeks—the third, or rheumatic stage coming on immediately after the eruptive. In general, however, the disease gave a degree of respite, for three, four, or even six weeks, when the joints became attacked with more pain and paralysis than at first—unaccompanied, however, by fever. These pains usually fixed themselves in one or two joints, and continued to excruciate the patient for weeks together. They were always severest in the morning, and wore off in some measure towards the evening. By the secondary pains many people were bed-ridden for days and weeks together, the slightest motion causing intense agony. Excepting these local pains, no other symptom of disease remained. The appetite was good, "and the unhappy patient, with most of the feelings of health about him, lay pinioned to the spot, like Gulliver, when he awoke on the shore of Lilliput." When the ma-

* See the notice of a peculiar epidemic in Paris, bearing a considerable analogy to the dandy fever.

lady had raged some time, and people were beginning to recover, it was amusing to see almost the whole population limping about, some with their arms in slings, others with their bodies half bent, and many on crutches.

The disease proved fatal in only three instances. The treatment was decidedly depletory, the lancet being the principal remedy. Dr. Stedman labours to prove that this peculiar epidemic was contagious; that it was *imported*, probably from Africa, into the Island of St. Thomas's, and was afterwards carried to Santa Cruz, a small island, lying 35 miles distant from St. Thomas's. We confess that we are not converts to the facts and arguments brought forwards by Dr. S. We firmly believe that the epidemic resulted from a peculiar miasm emanating from the earth, and widely diffused through the atmosphere. It will be seen that an epidemic, not very dissimilar, has raged in Paris during the last Summer, some account of which will be found in the present Number of our Journal. At the same time, we are not among those who refuse to admit a contagious character to a disease primarily atmospheric, or rather terrestrial. We are convinced that an epidemic may possess both characters.

---

